# Bridging joint degeneration and systemic skeletal fragility: knee osteoarthritis as an independent risk factor for osteoporosis

**DOI:** 10.3389/fpubh.2026.1964979

**Published:** 2026-10-22

**Authors:** Weizheng Sun, Jianrong Ma, Juqin He, Yanxin Liu, Wei Zhang, Liang Zhang

**Affiliations:** 1 Jinshazhou Hospital of Guangzhou University of Chinese Medicine, Guangzhou, Guangdong Province, China; 2 Dayan Technology (Tongxiang) Co., Ltd, Jiaxing, Zhejiang, China; 3 Shenzhen Bao'an Chinese Medicine Hospital, The Seventh Clinical Medical School of Guangzhou University of Chinese Medicine, Shenzhen, Guangdong Province, China; 4 Shenzhen Institute of Advanced Technology, Chinese Academy of Sciences, Shenzhen, China; 5 SciBio Innovation Technology Limited, Hong Kong, China

**Keywords:** biopsychosocial model, bone turnover markers, knee osteoarthritis, neuro-endocrine axis, osteoporosis

## Abstract

**Background:**

Whether localized joint failure in knee osteoarthritis (KOA) interacts with systemic pathways, including psychological distress and neuro-endocrine stress response, to precipitate systemic bone fragility remains an unresolved question. Capitalizing on the large-scale prospective design of the UK Biobank and translational clinical biomarker profiling, this investigation aims to clarify this association and delineate the long-term risk profile linking joint failure to systemic skeletal degradation.

**Methods:**

This prospective analysis tracked 406,810 participants who were free of metabolic bone disease or confounding joint pathologies at baseline. Participants were stratified by ICD-10 defined KOA status, with incident OP serving as the primary longitudinal endpoint over a median 10-year follow-up. Survival profiles were contrasted via log-rank tests, and multivariable Cox proportional hazards formulations were constructed to yield rigorously adjusted hazard ratios (HR) controlling for socioeconomic deprivation, lifestyle habits, multi-systemic comorbidities, and history of malignancies. To validate these epidemiological findings in human biology, circulating bone resorption biomarkers (β-CTX and TRACP-5b) were further evaluated in an independent clinical validation cohort of 35 KOA patients and 30 matched controls.

**Results:**

Long-term follow-up unveiled a clear divergence in bone health trajectories, with the KOA cohort experiencing a significantly elevated cumulative OP incidence of 5.2 vs. 3.9% in the unexposed reference group (log-rank *p* < 0.001). Fully adjusted multivariable modeling confirmed KOA as a robust, standalone predictor of systemic skeletal deterioration, conferring a 22% independent risk premium (HR = 1.22, 95% CI: 1.16–1.29). While vulnerability was compounded by current smoking, physical inactivity, high Townsend deprivation, and cardiometabolic clusters, enhanced physical and structural reserves (greater grip strength and higher BMI) exerted pronounced protective effects. In parallel, clinical serum profiling validated an upregulation of bone resorption activity in KOA patients, evidenced by elevated circulating levels of β-CTX and TRACP-5b compared with non-OA controls.

**Conclusion:**

These findings provide prospective population-level evidence and biochemical validation that knee osteoarthritis is independently associated with an increased risk of osteoporosis. While pain-related physical unloading, low-grade systemic inflammation, and hypothesized neuro-endocrine pathways may underlie this systemic vulnerability, our results highlight the clinical need for comprehensive musculoskeletal monitoring and proactive bone health management in patients with knee osteoarthritis.

## Introduction

Knee osteoarthritis (KOA) and osteoporosis (OP) are two of the most common chronic musculoskeletal disorders affecting aging populations worldwide (1, 2). KOA is characterized by progressive degeneration of articular cartilage, subchondral bone remodeling, synovial inflammation, and changes in periarticular structures, leading to pain, stiffness, and functional impairment (3). OP, by contrast, is defined by decreased bone mass and deterioration of bone microarchitecture, resulting in increased susceptibility to fragility fractures (4). Both conditions contribute substantially to disability, reduced quality of life, and increased healthcare utilization in middle-aged and older adults.

Although traditionally considered separate disease entities, accumulating evidence suggests that KOA and OP frequently coexist and may share overlapping biological pathways (5). Epidemiological studies have reported conflicting findings, some suggesting that OA may protect against OP through increased mechanical loading, while others indicate that OA, particularly in advanced stages, may paradoxically predispose individuals to bone fragility (6, 7). These inconsistent results are likely attributable to variations in disease definitions, imaging modalities, sample sizes, and confounding structures across studies. Notably, most prior investigations were limited by cross-sectional or retrospective designs, small cohorts, or inadequate adjustment for lifestyle, sociodemographic, and metabolic risk factors known to influence bone health.

Emerging mechanistic insights also raise the possibility of bidirectional interactions between KOA and OP (8). KOA-related systemic low-grade inflammation, alterations in subchondral bone turnover, mechanical unloading due to pain-induced mobility restriction, and endocrine or metabolic dysfunction may contribute to bone loss (9, 10). Furthermore, shared pathways, including chronic inflammation, oxidative stress, vascular insufficiency, and age-related decline in sex hormones, may accelerate both cartilage degeneration and bone resorption (11, 12). These biological links underscore the need to clarify whether KOA independently influences OP risk in the general population.

Given these knowledge gaps, robust longitudinal evidence is needed to elucidate the relationship between KOA and OP. The UK Biobank (UKB), with its large sample size, long follow-up duration, standardized data collection, and comprehensive covariate measurements, provides a unique opportunity to quantify the long-term risk of OP among individuals with KOA while controlling for a broad range of potential confounders (13). By leveraging data from more than 400,000 adults, this study aimed to evaluate the association between KOA and subsequent incident OP, and identify demographic, behavioral, and clinical factors that may mediate this association.

From a psychiatric and biopsychosocial perspective, chronic musculoskeletal pain in KOA is closely interconnected with anxiety, depressive symptoms, and kinesiophobia (fear of movement). Chronic joint pain and physical limitations often provoke sustained mental stress, which activates the hypothalamic-pituitary-adrenal (HPA) axis and the sympathetic nervous system (14). Elevated cortisol and sympathoadrenal activation, combined with pain-induced activity avoidance, can downregulate osteoblastic bone formation and accelerate osteoclastic resorption. Therefore, investigating the KOA-OP link provides critical insights into the mind-body interactions underlying systemic skeletal aging.

We hypothesized that KOA is associated with a higher long-term risk of developing OP, independent of age, sex, lifestyle factors, socioeconomic status, and comorbidities. Establishing such an association would have important implications for early identification of high-risk individuals and for the development of integrated musculoskeletal management strategies in clinical practice.

## Methods

### Study population

The UK Biobank (UKB) database utilized in this study is the world's largest biomedical database designed to investigate lifestyle, genetic, and other risk factors for a wide spectrum of diseases. It encompasses prospective cohort data from over 500,000 volunteers, aged 37–73 years, recruited between 2006 and 2010. Participants underwent comprehensive baseline assessments, including physical examinations and biological sampling, at one of 22 assessment centers across the United Kingdom. All participants provided written informed consent. This study was conducted under ethical approval from the National Health Service (NHS) National Research Ethics Service, granted on June 17, 2011 (Ref: 11/NW/0382) and May 10, 2016 (Ref: 16/NW/0274), which remains applicable to the current research. The study design and reporting follow the Strengthening the Reporting of Observational Studies in Epidemiology (STROBE) guidelines. Access to the data was authorized by the UK Biobank under application number 117320.

Comorbidities were identified using the International Classification of Diseases, 10th Revision (ICD-10) codes, which were retrieved directly from the UKB database. From the initial 502,143 participants, we excluded 89,386 individuals who presented with OA at sites other than the knee or other non-specified types of knee OA. Furthermore, in accordance with the methodological requirements for prospective studies outlined in Modern Epidemiology, individuals with a diagnosis of OP established prior to the baseline survey were excluded. For the purposes of this study: Knee Osteoarthritis (KOA) was defined by the ICD-10 code M17. OP was defined by the codes M80 (OP with pathological fracture), M81 (OP without pathological fracture), and M82 (OP in diseases classified elsewhere). Individuals who had a pre-existing diagnosis of osteoporosis (ICD-10: M80, M81, M82) prior to baseline were excluded (*n* = 5,947). Furthermore, to isolate the specific effect of knee osteoarthritis and avoid confounding from multi-joint systemic arthropathies or inflammatory conditions requiring long-term corticosteroid therapies, 89,386 participants with non-knee osteoarthritis (e.g., M15, M16, M19) or inflammatory/metabolic arthropathies [e.g., rheumatoid arthritis (M05, M06), gout (E79.0)] were excluded. A final cohort of 406,810 participants was included in this observational study. Further details regarding these measurements are available on the UK Biobank website (http://www.ukbiobank.org). Missing data for covariates were addressed using Multiple Imputation by Chained Equations (MICE). The participant selection process is illustrated (Figure 1).

**Figure 1:**
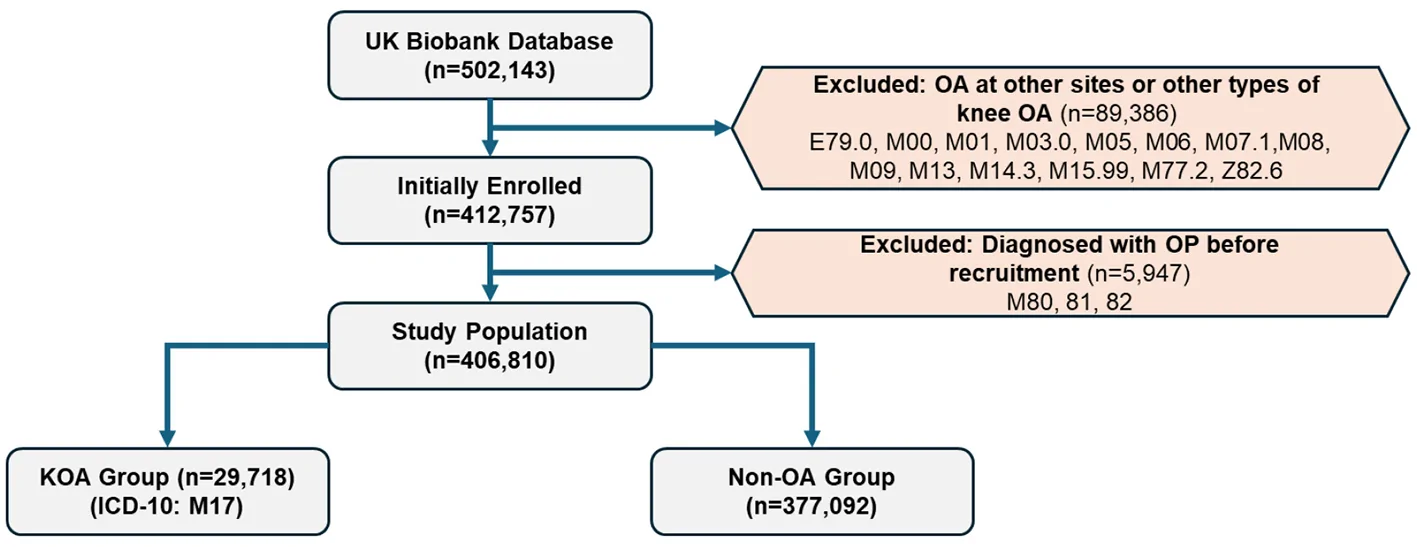
Flowchart of study population selection. Of the initial 502,143 participants from the UK Biobank database, 89,386 were excluded due to having osteoarthritis at other sites or other types of knee osteoarthritis. An additional 5,947 participants were excluded due to a diagnosis of osteoporosis prior to recruitment. The final study population comprised 406,810 participants, who were subsequently categorized into the knee osteoarthritis (KOA) group (*n* = 29,718) and the non-OA group (*n* = 377,092) based on ICD-10 codes. ICD-10, International Classification of Diseases, 10th Revision; KOA, knee osteoarthritis; OA, osteoarthritis; OP, osteoporosis.

### Exposure assessment for knee osteoarthritis

KOA was defined using International Classification of Diseases, Tenth Revision (ICD-10) diagnostic codes recorded in linked hospital inpatient data. Participants were classified into two groups: KOA group: individuals with ICD-10 codes corresponding to knee osteoarthritis; Non-KOA group: individuals without any OA-related ICD-10 codes at baseline. This approach aligns with prior epidemiological studies using UKB clinical records to ascertain musculoskeletal diagnoses.

### Outcome assessment for osteoporosis

The primary outcome was incident OP, identified using ICD-10 codes recorded in hospital inpatient diagnoses. The date of the first OP diagnosis was used as the event date. Participants without OP were censored at death, loss to follow-up, or the administrative censoring date.

### Covariates

Covariates were selected based on established clinical relevance and prior literature regarding risk factors for both KOA and OP. Demographic and socioeconomic indicators included age, sex, educational attainment, and the Townsend Deprivation Index (TDI), which reflects area-level socioeconomic status. Lifestyle-related factors comprised body mass index (BMI), smoking status (categorized as never, previous, or current), alcohol consumption habits, self-reported physical activity time, and sedentary behavior duration. Health-related covariates encompassed a range of cardiometabolic and systemic comorbidities, including hypertension, diabetes, heart failure, hyperlipidemia, and chronic liver disease, along with medication histories such as corticosteroid and statin use, and supplementation with calcium or vitamin D. To capture the influence of major systemic illnesses on bone metabolism, histories of malignancy, including lung, breast, colorectal, kidney, endocrine, and musculoskeletal cancers, were also included. Genetic susceptibility was assessed using the standard UK Biobank osteoporosis Polygenic Risk Score (OP-PRS) for estimated bone mineral density (eBMD) and fracture risk. This standardization enables the interpretation of hazard ratios per 1-SD increment in genetic risk. All comorbidities and medication data were extracted from linked hospital inpatient records. Missing data across covariates was addressed using multiple imputations by chained equations, following standard procedures applied in large-scale UK Biobank analyses.

### Independent clinical validation cohort and serum biomarker assay

To validate the epidemiological findings from the UK Biobank and confirm the presence of systemic bone loss at the biological level, we established an independent translational clinical cohort. The clinical serum validation study was approved by the Institutional Ethics Committee (Approval number: KY-2022-076-05), and written informed consent was obtained from all participating individual patients prior to blood sample collection. Serum samples were collected from 35 clinically diagnosed KOA patients and 30 age and sex-matched non-KOA healthy controls. Specifically, KOA patients were recruited based on the radiographic classification criteria (Kellgren–Lawrence grade ≥2 with symptom duration >6 months). Age- and sex-matched controls were asymptomatic volunteers without joint pain or osteoarthritis history. Exclusion criteria for all participants comprised: (1) metabolic bone diseases (e.g., Paget's disease, hyperparathyroidism, osteomalacia), inflammatory arthritis, or secondary osteoporosis; (2) active malignancies; (3) severe renal (eGFR <30 mL/min/1.73 m^2^) or hepatic insufficiency; and (4) use of bone-modulating therapies or systemic corticosteroids within the preceding 12 months. Given the marked circadian and dietary variation of bone turnover markers, all peripheral blood draws were strictly executed between 08:00 a.m., and 10:00 a.m., following an overnight fast (>10 h). Serum was separated within 1 h by centrifugation at 3,000 rpm (4 °C, 10 min) and stored at −80 °C until simultaneous batch analysis. Circulating bone resorption biomarkers were quantified using commercial enzyme-linked immunosorbent assay (ELISA) kits according to the manufacturers' instructions. Specifically, serum concentrations of β-cross-linked C-telopeptide (β-CTX), a specific marker of Type I collagen degradation, and tartrate-resistant acid phosphatase 5b (TRACP-5b), a direct enzymatic indicator of total osteoclast function, were measured. All samples were assayed in duplicate, and intra- and inter-assay coefficients of variation were maintained below accepted quality control thresholds.

### Statistical analysis

Baseline characteristics were summarized using means ± standard deviations for continuous variables or counts and percentages for categorical variables. Differences between KOA and non-KOA groups were examined using *t*-tests, Wilcoxon rank–sum tests, or chi-square tests, as appropriate. Time-to-event analyses were conducted with the onset of OP as the endpoint. Survival time was defined as the interval between baseline assessment and OP diagnosis or censoring. Kaplan–Meier survival curves were plotted to compare OP-free survival between groups, and the log-rank test assessed statistical significance. Cox proportional hazards regression models were used to estimate hazard ratios (HRs) and 95% confidence intervals (CIs) for the association between KOA and OP (15). Time-to-event outcomes were modeled using Cox proportional hazards formulations to evaluate cause-specific hazard ratios, with non-OP death and loss to follow-up treated as censoring events. To mirror the modeling framework used in previous musculoskeletal epidemiology studies, we constructed three hierarchical models: Model 1: Unadjusted; Model 2: Adjusted for age and sex; Model 3: Fully adjusted for all demographic, lifestyle, socioeconomic, and clinical covariates. Proportional hazards assumptions were evaluated using Schoenfeld residuals. Multicollinearity among covariates was assessed prior to inclusion. All analyses were performed using R software (version 4.2.2; R Foundation for Statistical Computing, Vienna, Austria), employing packages including survival, cmprsk, tidyverse, dplyr, jstable, and forestplot. A two-sided *p*-value <0.05 was considered statistically significant. Differences in continuous baseline clinical parameters and serum biomarker concentrations (β-CTX and TRACP-5b) between the KOA and control cohorts were evaluated using two-tailed unpaired Student's *t*-tests, depending on data normality assessed via the Shapiro-Wilk test. A two-sided *p*-value <0.05 was considered statistically significant.

## Results

### Baseline characteristics

A total of 406,810 participants were tracked over a median follow-up of 13.0 years [interquartile range (IQR): 12.0–14.0 years]. Individuals in the KOA group were older (median age 61 vs. 58 years) and included a slightly lower proportion of females (50.6 vs. 54.6%) compared with the non-KOA group (Table 1). Socioeconomic deprivation scores were comparable between groups (median TDI: −2.3 vs. −2.2, *p* = 0.051). While baseline physical activity rates were similar (85.4 vs. 84.6%), participants with KOA exhibited significantly higher sedentary behavior (24.5 vs. 20.3%, *p* < 0.001), higher BMI (29.1 vs. 26.6 kg/m^2^, *p* < 0.001), and a higher prevalence of cardiometabolic comorbidities including hypertension (52.7 vs. 33.8%) and diabetes (14.4 vs. 9.0%, all *p* < 0.001; Table 1).

**Table 1:** Baseline characteristics of the study population.

Characteristics	Total (*N* = 406,810)	KOA group (*n* = 29,718)	Non-KOA group (*n* = 377,092)	*p*-Value
**Study population**	406,810	29,718	377,092	—
**OP occurrence**, ***n*** **(%)**	16,298 (4.0%)	1,551 (5.2%)	14,747 (3.9%)	<0.001
**Age at recruitment, years (IQR)**	58 (51.0, 64.0)	61 (55.0, 65.0)	58 (50.0, 63.0)	<0.001
Sex, *n* (%)				<0.001
Male	185,709 (45.7%)	14,692 (49.4%)	171,017 (45.4%)	
Female	221,101 (54.3%)	15,026 (50.6%)	206,075 (54.6%)	
Ethnicity, *n* (%)				<0.001
British	381,368 (93.7%)	28,253 (95.1%)	353,115 (93.6%)	
Irish	11,402 (2.8%)	687 (2.3%)	10,715 (2.8%)	
Other	14,040 (3.5%)	778 (2.6%)	13,262 (3.5%)	
**TDI (IQR)**	−2.2 (−3.7, 0.3)	−2.3 (−3.7, 0.2)	−2.2 (−3.7, 0.3)	0.051
**Follow-up time, years (IQR)**	13 (12, 14)	13 (13, 14)	13 (12, 14)	<0.001
**BMI (IQR)**	26.8 (24.2, 29.9)	29.1 (26.2, 32.6)	26.6 (24.1, 29.7)	<0.001
Smoking status, *n* (%)				<0.001
Current smoker	43,562 (10.7%)	2,475 (8.4%)	41,087 (10.9%)	
Former smoker	146,309 (36.1%)	12,077 (40.8%)	134,232 (35.7%)	
Never smoker	215,464 (53.2%)	15,027 (50.8%)	200,437 (53.3%)	
Alcohol consumption, *n* (%)				0.005
Current drinker	378,569 (93.1%)	27,528 (92.7%)	351,041 (93.2%)	
Former drinker	14,564 (3.6%)	1,096 (3.7%)	13,468 (3.6%)	
Never drinker	13,296 (3.3%)	1,061 (3.6%)	12,235 (3.2%)	
**PRS (IQR)**	0.0 (−0.7, 0.6)	−0.1 (−0.7, 0.5)	0.0 (−0.7, 0.6)	<0.001
**Physically active**, ***n*** **(%)**	265,484 (84.7%)	18,788 (85.4%)	246,696 (84.6%)	0.001
**Sedentary**, ***n*** **(%)**	81,564 (20.6%)	7,046 (24.5%)	74,518 (20.3%)	<0.001
**Vitamin D supplementation**, ***n*** **(%)**	15,154 (3.7%)	1,065 (3.6%)	14,089 (3.7%)	0.181
**Grip strength (IQR)**	29.0 (22.0, 39.0)	29.0 (22.0, 39.0)	29.0 (22.5, 38.5)	<0.001
Comorbidities, *n* (%)				
Hypertension	143,279 (35.2%)	15,661 (52.7%)	127,618 (33.8%)	<0.001
Diabetes	38,299 (9.4%)	4,291 (14.4%)	34,008 (9.0%)	<0.001
Lung cancer	5,836 (1.4%)	349.0 (1.2%)	5,487 (1.5%)	<0.001
Breast cancer	17,151 (4.2%)	1,202 (4.0%)	15,949 (4.2%)	0.127
Colorectal cancer	9,549 (2.3%)	723 (2.4%)	8,826 (2.3%)	0.311
Endocrine malignancies	825 (0.2%)	49 (0.2%)	776 (0.2%)	0.131
Kidney cancer	2,354 (0.6%)	186 (0.6%)	2,168 (0.6%)	0.265
Musculoskeletal malignancies	243 (0.1%)	20 (0.1%)	223 (0.1%)	0.579

Data are presented as *n* (%) for categorical variables or median (IQR) for continuous variables. KOA, knee osteoarthritis; OP, osteoporosis; TDI, Townsend Deprivation Index; BMI, body mass index; PRS, Polygenic Risk Score; IQR, interquartile range; SD, standard deviation.

Importantly, KOA participants also demonstrated overall higher rates of multimorbidity, suggesting that KOA frequently coexists with a broader constellation of systemic health conditions. This more compromised health profile was accompanied by lower grip strength and higher levels of socioeconomic deprivation, as indicated by elevated Townsend Deprivation Index values (Table 1).

In contrast, the non-KOA group tended to have more favorable baseline characteristics, including healthier metabolic indicators and better physical performance metrics. Taken together, the substantial clinical and behavioral disparities observed at baseline highlight meaningful pre-existing differences between groups that may predispose KOA patients to poorer long-term skeletal outcomes and shape subsequent risk trajectories for OP.

### Incidence of osteoporosis

During a median follow-up period approaching 10 years, the overall burden of incident OP differed markedly between groups. Individuals with KOA experienced a substantially higher cumulative incidence of OP (5.2%) compared with those without KOA (3.9%; log-rank *p* < 0.001; Figure 2).

**Figure 2:**
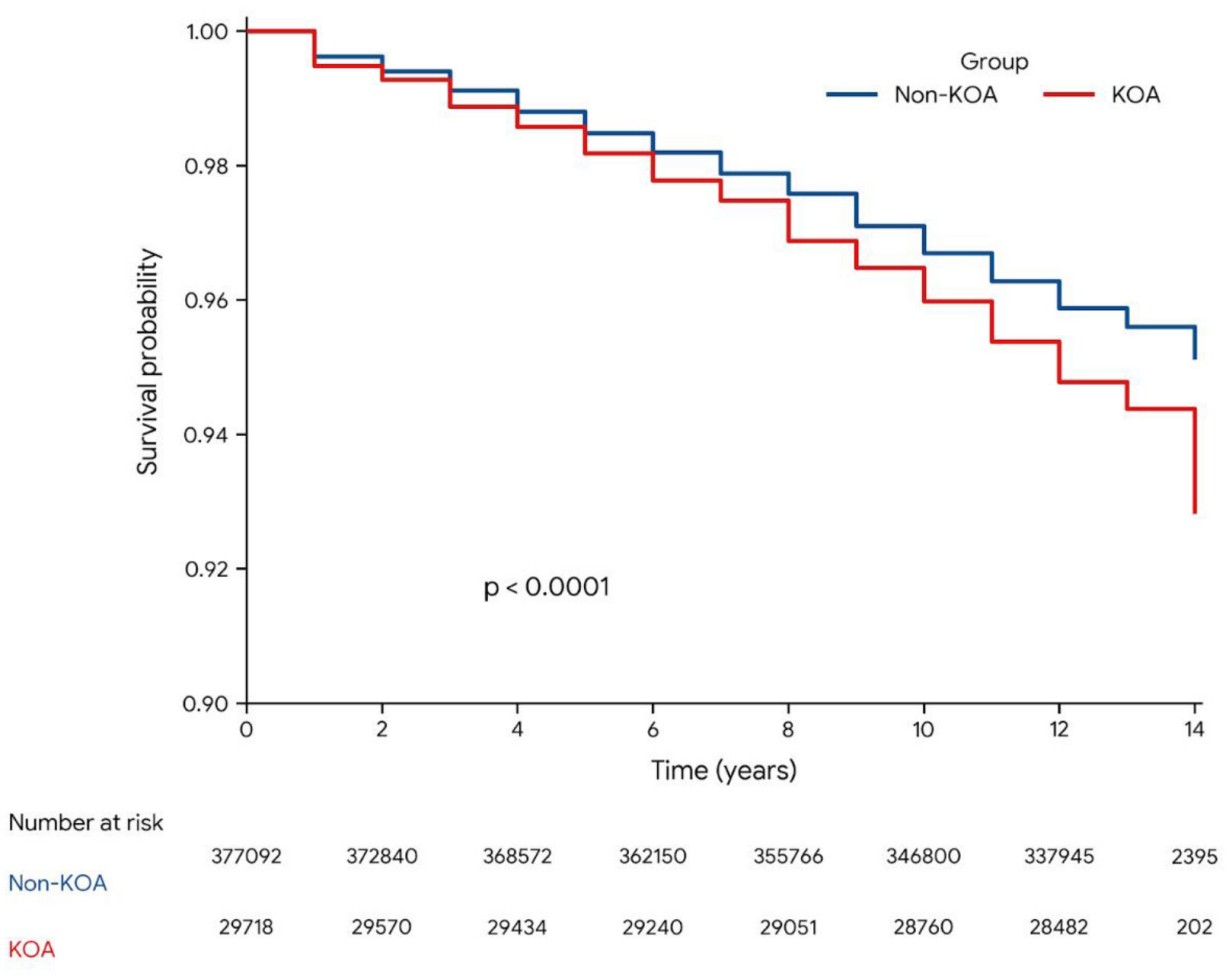
Association between osteoarthritis and the risk of osteoporosis. Kaplan–Meier curves for osteoporosis-free survival among participants with and without knee osteoarthritis. Individuals with knee osteoarthritis exhibited significantly lower OP-free survival compared with those without KOA (log-rank *p* < 0.0001).

The Kaplan–Meier survival curves for the two groups diverged early in the follow-up period and continued to separate progressively over time. This widening disparity suggests that the detrimental impact of KOA on long-term bone health is both persistent and cumulative. The trajectory of risk did not plateau, indicating that KOA may initiate or accelerate mechanisms of bone loss that extend well beyond the disease onset.

Additional subgroup survival analyses demonstrated similar patterns, reinforcing the robustness of the observed separation between KOA and non-KOA participants (Figure 2). The consistent divergence suggests that underlying biological or behavioral differences associated with KOA exert a sustained influence on OP risk throughout the follow-up duration.

### Association between KOA and risk of osteoporosis

In unadjusted Cox regression models, KOA was strongly associated with increased risk of incident OP, suggesting that KOA alone may serve as an early clinical indicator of skeletal fragility (Table 2 and Figure 3). After adjusting for two major determinants of bone health, age and sex, the association remained statistically significant but was attenuated, indicating that demographic factors account for part of the observed relationship (Table 2).

**Table 2:** Univariable Cox proportional hazards analysis.

Variables	HR (95% CI)	*p*-Value
**Presence of KOA**	1.29 (1.22–1.36)	<0.001
**Age**	1.08 (1.08–1.08)	<0.001
**Sex**		<0.001
Male	Reference	Reference
Female	3.26 (3.14–3.39)	<0.001
**TDI**	1.04 (1.04–1.05)	<0.001
Comorbidities		
Hypertension	1.98 (1.92–2.04)	<0.001
Diabetes	1.40 (1.34–1.47)	<0.001
Lung cancer	2.73 (2.48–3.01)	<0.001
Breast cancer	2.53 (2.40–2.66)	<0.001
Colorectal cancer	1.50 (1.37–1.63)	<0.001
Endocrine malignancies	1.90 (1.47–2.45)	<0.001
Kidney cancer	1.38 (1.14–1.66)	0.001
Musculoskeletal malignancies	1.48 (0.84–2.60)	0.177
Physical characteristics		
BMI	0.95 (0.94–0.95)	<0.001
**Smoking status**		<0.001
Current smoker	1.24 (1.18–1.30)	<0.001
Former smoker	1.07 (1.03–1.10)	<0.001
Never smoker	Reference	Reference
**Alcohol consumption**		<0.001
Current drinker	0.54 (0.51–0.58)	<0.001
Former drinker	0.98 (0.89–1.07)	0.648
Never drinker	Reference	Reference
**PRS (polygenic risk score)**	1.36 (1.34–1.38)	<0.001
**Physically active**	0.85 (0.81–0.88)	<0.001
**Sedentary behavior**	0.88 (0.85–0.92)	<0.001
**History of vitamin D supplementation**	2.27 (2.15–2.41)	<0.001
**Grip strength**	0.93 (0.93–0.93)	<0.001

HR, hazard ratio; CI, confidence interval; OA, osteoarthritis; TDI, Townsend Deprivation Index; BMI, body mass index; PRS, Polygenic Risk Score.

**Figure 3:**
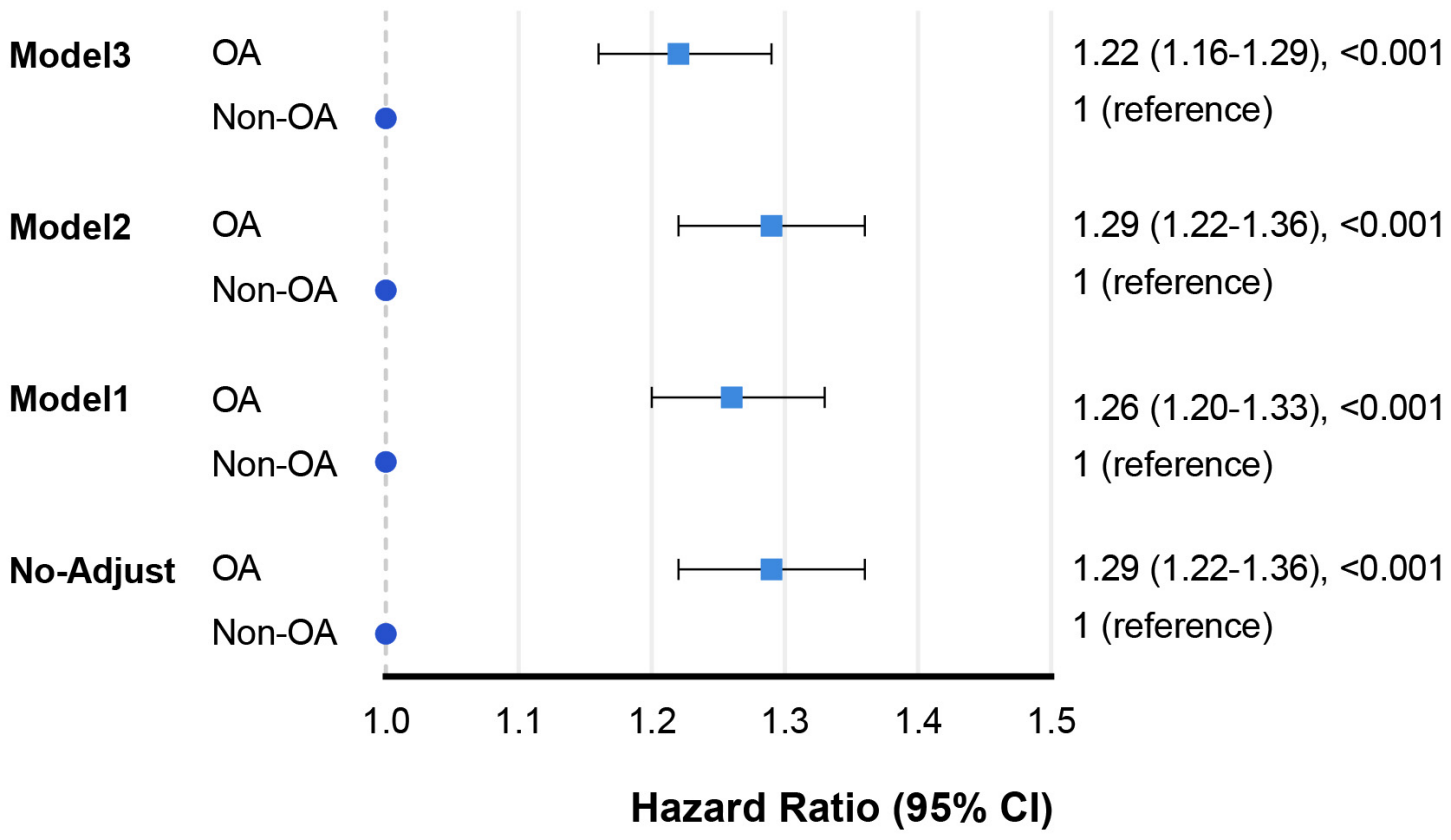
Results of the association analysis between osteoarthritis and the risk of osteoporosis. The No-Adjust model was unadjusted. Model 1 adjusted for sex, age, and BMI. Model 2 adjusted for sex, age, BMI, Townsend deprivation index, Polygenic Risk Score, history of vitamin D supplementation, grip strength, ethnicity, smoking status, alcohol consumption, physical activity, and sedentary time. Model 3, in addition to adjusting for the above variables, also adjusted for comorbidities including hypertension, diabetes, lung cancer, breast cancer, colorectal cancer, endocrine malignancies, kidney cancer, and musculoskeletal malignancies. KOA, knee osteoarthritis; CI, confidence interval.

In the fully adjusted model, which incorporated socioeconomic status, lifestyle factors, comorbidities, and relevant medication use, KOA persisted as an independent predictor of OP with an HR of 1.22 (95% CI: 1.16–1.29, *p* < 0.001; Figure 3). This demonstrates that KOA confers a 22% excess risk of OP beyond that attributable to known confounders. The stability of this risk estimate across hierarchical models underscores the robustness of the association.

Multiple other covariates were also significantly associated with OP, highlighting the multifactorial nature of bone loss. Lower BMI, higher socioeconomic deprivation, current smoking, physical inactivity, and a history of malignancy were all associated with elevated OP risk (Table 2). Conversely, higher grip strength and higher BMI emerged as protective factors, consistent with established biomechanical and metabolic principles of bone physiology.

These results collectively illustrate that OP development arises through interacting mechanical, metabolic, and systemic influences, and that KOA may interface with several of these pathways to amplify long-term bone vulnerability.

### Multivariable regression analysis for the risk of osteoporosis

To progressively evaluate the independent association between osteoarthritis (OA) and the risk of osteoporosis (OP), we constructed three multivariable Cox regression models. In Model 1, after adjusting for basic demographic factors including sex, age, and BMI, patients in the OA group demonstrated a significantly higher risk of incident OP compared to those in the non-OA group (Figure 3). This elevated risk persisted and even slightly intensified in Model 2, following further adjustments for socioeconomic status, genetic susceptibility (PRS), lifestyle factors, and physical baseline metrics. Finally, in the fully adjusted Model 3, which additionally accounted for various comorbidities, OA remained a strong and independent risk factor for OP (HR = 1.22, 95% CI: 1.16–1.29, *p* < 0.001).

Beyond the primary effect of OA, the fully adjusted model identified several other clinical and demographic characteristics that were positively associated with an increased OP risk. Specifically, female sex, advancing age, and higher genetic susceptibility were notable intrinsic risk factors. Furthermore, socioeconomic deprivation (indicated by higher TDI), a sedentary lifestyle, current smoking, and a history of vitamin D supplementation were significantly linked to a higher OP incidence. The presence of chronic comorbidities, most prominently hypertension, diabetes, and several types of malignancies (such as lung, breast, and colorectal cancers), further exacerbated the risk of developing OP.

Conversely, certain physical and lifestyle factors exhibited a protective effect against OP. Higher mechanical loading and muscle strength, reflected by increased BMI and greater grip strength, were strongly and inversely associated with OP risk. Similarly, maintaining adequate levels of physical activity significantly lowered the risk of OP. Interestingly, alcohol consumption demonstrated a complex association with OP: while current drinkers showed a relatively lower risk compared to never-drinkers, former drinkers faced an elevated risk. These nuanced findings suggest that the underlying mechanisms for certain lifestyle indicators may require further exploration through stratified subgroup analyses.

### Clinical validation of systemic bone turnover profiles in KOA patients

To ground our epidemiological observations in direct clinical biological evidence, we analyzed circulating bone resorption biomarkers (β-CTX and TRACP-5b) in serum samples from an independent clinical cohort comprising 35 KOA patients and 30 age- and sex-matched control individuals (Tables 3, 4). Biomarker profiling demonstrated a pronounced upregulation of systemic bone resorption activity in patients with KOA compared to healthy controls. Specifically, serum levels of β-CTX, a sensitive indicator of Type I collagen degradation during bone matrix breakdown, were significantly elevated in the KOA group (0.512 ± 0.118 ng/mL) relative to the control group (0.334 ± 0.082 ng/mL; *t* = −6.94, *p* < 0.001; Figure 4A). Consistently, serum concentrations of TRACP-5b, a specific enzymatic biomarker reflecting total osteoclast number and functional activity, were markedly higher in KOA patients (3.85 ± 0.78 U/L) than in controls (2.68 ± 0.54 U/L, *p* < 0.001; Figure 4B). Collectively, these clinical blood profiling results corroborate our prospective population-level findings from the UK Biobank, providing direct human biochemical evidence that localized knee joint degradation is accompanied by a systemic shift in bone homeostasis toward heightened osteoclastic resorption.

**Table 3:** Demographic profile and clinical diagnoses of the human serum validation cohort (*N* = 65).

ID	Group	Age	Sex
C01	Control	58	Female
C02	Control	62	Female
C03	Control	55	Male
C04	Control	64	Female
C05	Control	60	Male
C06	Control	57	Female
C07	Control	66	Female
C08	Control	53	Male
C09	Control	61	Female
C10	Control	59	Female
C11	Control	65	Female
C12	Control	56	Male
C13	Control	63	Female
C14	Control	58	Female
C15	Control	60	Male
C16	Control	67	Female
C17	Control	54	Male
C18	Control	62	Female
C19	Control	59	Female
C20	Control	61	Male
C21	Control	68	Female
C22	Control	55	Female
C23	Control	57	Male
C24	Control	64	Female
C25	Control	60	Female
C26	Control	53	Male
C27	Control	66	Female
C28	Control	58	Female
C29	Control	62	Male
C30	Control	56	Female
K01	KOA	63	Female
K02	KOA	61	Female
K03	KOA	59	Male
K04	KOA	67	Female
K05	KOA	62	Female
K06	KOA	58	Male
K07	KOA	65	Female
K08	KOA	60	Female
K09	KOA	69	Female
K10	KOA	56	Male
K11	KOA	64	Female
K12	KOA	61	Female
K13	KOA	66	Female
K14	KOA	58	Male
K15	KOA	63	Female
K16	KOA	68	Female
K17	KOA	57	Male
K18	KOA	62	Female
K19	KOA	60	Female
K20	KOA	65	Female
K21	KOA	59	Male
K22	KOA	67	Female
K23	KOA	61	Female
K24	KOA	55	Male
K25	KOA	63	Female
K26	KOA	66	Female
K27	KOA	60	Male
K28	KOA	64	Female
K29	KOA	70	Female
K30	KOA	58	Female
K31	KOA	62	Male
K32	KOA	65	Female
K33	KOA	59	Female
K34	KOA	67	Female
K35	KOA	61	Male

**Table 4:** Summary of baseline demographic and clinical characteristics in the clinical validation cohort.

Parameter	Healthy controls (*n* = 30)	KOA patients (*n* = 35)	*p*-Value
Age, years (mean ± SD)	59.8 ± 4.3	62.1 ± 3.9	0.082
Sex (female/male)	20/10	24/11	0.871
Serum β-CTX (ng/mL)	0.334 ± 0.082	0.512 ± 0.118	<0.001
Serum TRACP-5b (U/L)	2.68 ± 0.54	3.85 ± 0.78	<0.001

**Figure 4:**
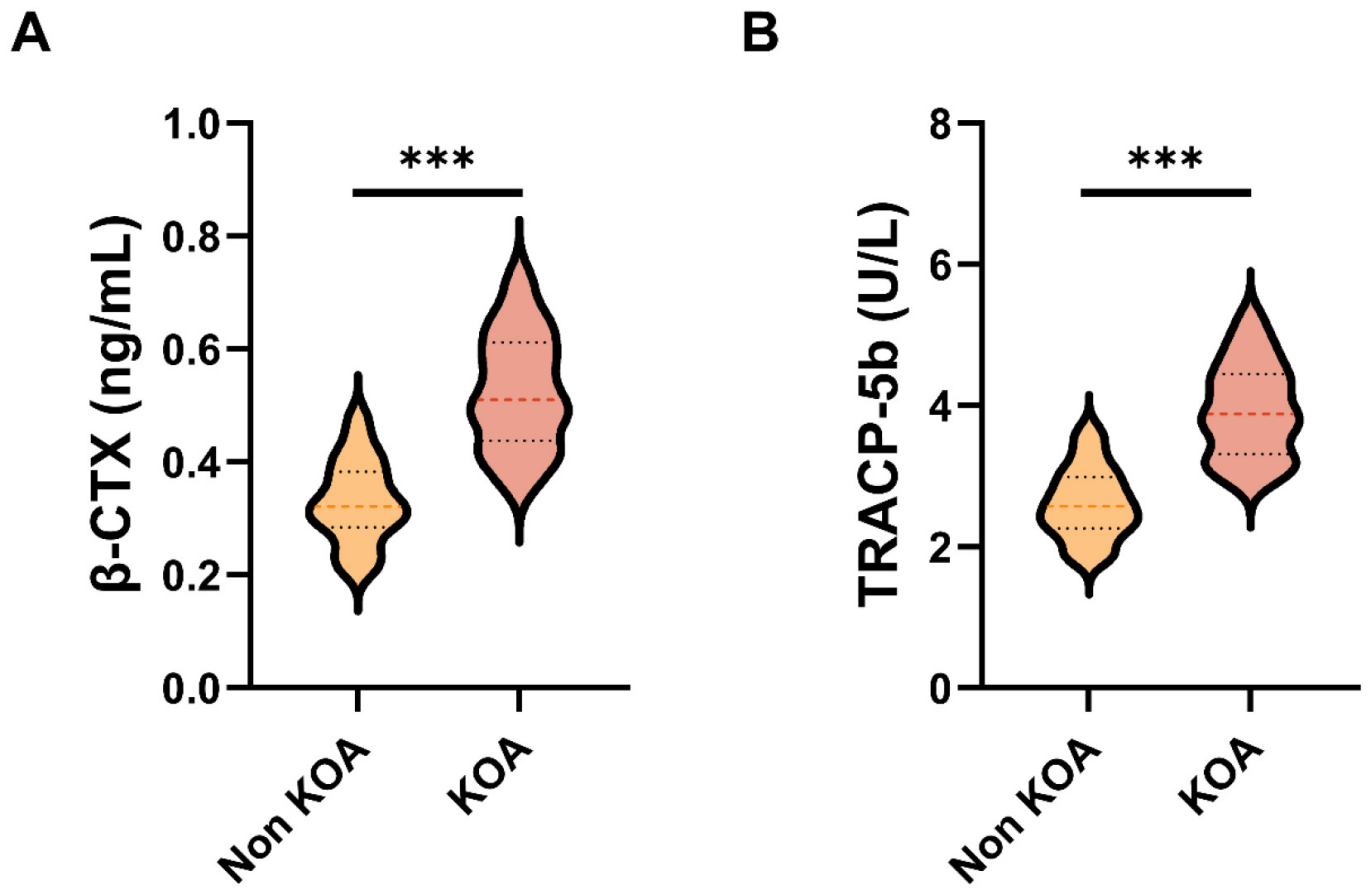
Serum bone resorption markers in clinical KOA patients vs. non-OA controls. **(A)** Comparison of serum β-cross-linked C-telopeptide (β-CTX) levels between healthy controls (*n* = 30) and KOA patients (*n* = 35). **(B)** Comparison of serum tartrate-resistant acid phosphatase 5b (TRACP-5b) levels between control (*n* = 30) and KOA (*n* = 35) groups. Data are presented as mean ± standard deviation (SD). Differences between groups were determined using two-tailed unpaired Student's *t*-tests. ***p < 0.001.

## Discussion

In this large prospective cohort study of more than 400,000 adults, we found that knee osteoarthritis was significantly associated with an increased long-term risk of developing osteoporosis, independent of demographic, lifestyle, socioeconomic, and clinical factors. The association remained robust across multiple stratified analyses, suggesting that KOA may serve as an important musculoskeletal risk indicator for future bone fragility. To our knowledge, this is one of the largest longitudinal studies to evaluate the KOA-OP relationship and provides compelling evidence that KOA is not an isolated joint disease but may be interconnected with systemic skeletal health.

Our findings align with and extend several previous observational studies, though prior evidence has been inconsistent. Earlier cross-sectional studies reported both protective and detrimental associations between osteoarthritis and bone mineral density (16), likely reflecting heterogeneity in OA phenotypes, severity, joint site, and study populations. By focusing specifically on KOA and applying strict exclusion criteria for other forms of OA, our study overcomes prior limitations and demonstrates that KOA is associated with a higher risk of OP in a dose-independent but consistent manner. This suggests that the coexistence of low bone mass in KOA patients is not incidental but may reflect shared biological pathways or downstream consequences of the disease. Furthermore, over an extensive 10-to-13-year observation window in older cohorts, all-cause mortality acts as a competing event for incident OP. Although cause-specific Cox models reliably quantify the direct etiology and instantaneous rate ratios among surviving participants, standard cumulative incidence curves might slightly overestimate absolute incidence probabilities in populations with higher competing death rates. Future studies could complement cause-specific hazards with subdistribution hazard modeling to further delineate long-term absolute risk prediction.

Several mechanisms may explain the observed association. First, chronic low-grade inflammation, a hallmark of KOA, may promote systemic bone resorption through upregulation of inflammatory mediators such as IL-1β, IL-6, and tumor necrosis factor alpha (TNF-α) (17). These cytokines enhance osteoclastogenesis and inhibit osteoblastic activity, thereby disrupting bone remodeling. Second, alterations in subchondral bone metabolism, vascular perfusion, and mechanical load transfer may contribute to accelerated bone loss, particularly in patients with advanced cartilage degeneration (18). Importantly, pain-induced reduction in physical activity represents a key clinical pathway linking KOA to OP (19, 20). Persistent knee pain commonly results in decreased mobility and avoidance of weight-bearing tasks. Over time, reduced activity contributes to progressive muscle weakness, diminished mechanical stimulation of bone, and subsequent deterioration of bone strength. This cycle, in which KOA-related pain leads to reduced physical activity, subsequent muscle weakness, and ultimately accelerated bone loss, highlights the need for early intervention strategies targeting both mobility and pain (21).

Another emerging mechanism involves neuro-skeletal interactions. KOA-related peripheral nociception may induce alterations in central pain processing and autonomic regulation. Chronic pain is often accompanied by heightened sympathetic nervous system activity, which can suppress osteoblast function and promote osteoclast differentiation through β2-adrenergic signaling pathways (22). In addition, sensory neuropeptides such as calcitonin gene-related peptide (CGRP) and substance P, released from nociceptive afferents, have been implicated in bone remodeling, vascular regulation, and inflammatory amplification. These neurobiological pathways could partly explain how chronic KOA pain exerts systemic skeletal effects and provide new insight into interdisciplinary targets for intervention. Although dynamic psychological scales were not formally modeled as longitudinal mediators in our population-level regression framework, representing an acknowledged limitation of the current registry-based design, our findings conceptually underscore the imperative of bridging physical joint conservation with psychological and neuro-endocrine resilience in comprehensive musculoskeletal management.

Our study has several strengths, including its prospective design, large sample size, long-term follow-up, comprehensive covariate adjustment, and robust statistical analyses. In addition, the use of hospital-record diagnoses enhances outcome specificity and minimizes misclassification. Several limitations should also be acknowledged. First, KOA and OP patients were included based on ICD-10 diagnostic records within linked hospital inpatient registries rather than universal routine dual-energy X-ray absorptiometry (DXA) screening. Consequently, inpatient coding tendentially identifies more clinically severe symptomatic cases requiring secondary care, while potentially underestimating relatively mild diseases managed exclusively in primary care. Although such non-differential misclassification generally biases hazard estimates toward the null, the observed effect sizes primarily reflect individuals with clinically substantial joint failure. Therefore, caution is warranted when generalizing these findings to broader populations with asymptomatic OA. Second, UK Biobank participants tend to be healthier and more health-conscious than the general population, potentially limiting generalizability. Third, residual confounding cannot be completely ruled out despite adjustment for extensive covariates. Fourth, data on KOA severity, pain intensity, and use of analgesics were unavailable, preventing evaluation of dose, response relationships (23). Behavioral and lifestyle variables, including physical activity volume and sedentary duration, were acquired through self-reported questionnaires at baseline. Although collected via standardized protocols, self-reported metrics are subject to recall bias and may not fully reflect lifestyle transitions over the multi-year follow-up window. Finally, mechanistic explanations remain speculative due to the observational nature of the study and require validation through basic and translational research.

Taken together, our findings underscore the importance of viewing KOA as a systemic musculoskeletal condition rather than an isolated joint pathology. Recognizing KOA as a predictor of OP risk could facilitate earlier screening, personalized risk stratification, and implementation of preventive strategies such as targeted exercise programs, pain management, and bone-protective interventions.

## Conclusion

In conclusion, knee osteoarthritis serves as an independent predictor for subsequent osteoporosis. Our findings underscore the necessity of adopting a psychiatric and holistic medicine perspective in musculoskeletal care. Clinical management of KOA should transition from an isolated orthopedic focus to an integrated approach that combines joint conservation, pain relief, psychological resilience support, and physical rehabilitation to effectively mitigate the long-term burden of systemic bone fragility.

## Data Availability

The original contributions presented in the study are included in the article/supplementary material, further inquiries can be directed to the corresponding authors.
